# A c-type lectin *HcLec1* with dual function of immunology and mineralization from the freshwater oyster (*Hyriopsis cumingii* Lea)

**DOI:** 10.3389/fimmu.2024.1530732

**Published:** 2025-01-14

**Authors:** Xiaofeng Chen, Xiaoya Shen, Shijun Liu, Wenjuan Li, He Wang, Jiale Li, Zhiyi Bai

**Affiliations:** ^1^ Key Laboratory of Freshwater Aquatic Genetic Resources, Ministry of Agriculture and Rural Affairs, Shanghai Ocean University, Shanghai, China; ^2^ Science and Technology Service Center, Shanghai MugaoBiotechnology co., Ltd., Shanghai, China; ^3^ Shanghai Engineering Research Center of Aquaculture, Shanghai Ocean University, Shanghai, China

**Keywords:** C-type lectin, immunity, biomineralization, calcite formation, bacterial agglutination, shell repair

## Abstract

**Background:**

Shell and pearl formation in bivalves is a sophisticated biomineralization process that encompasses immunological and mineralization aspects, particularly during shell repair and the initial stages of pearl cultivation when a nucleus is inserted. Here, we describe a novel C-type lectin, HcLec1, isolated and characterized from the freshwater pearl mussel *Hyriopsis cumingii* Lea.

**Methods:**

Immune challenge, RNA interference (RNAi) experiments, ELISA, and antibacterial assays were employed to investigate the role of HcLec1 in innate immunity. We also established shell damage repair and pearl nucleus insertion models to examine the impact of *HcLec1* on the biomineralization process in *Hyriopsis cumingii* Lea. In vitro calcium carbonate crystallization assays were conducted to explore the direct role of *HcLec1* in calcium carbonate crystal formation.

**Results:**

The *HcLec1* gene sequence is a full-length cDNA of 1552 bp, encoding 240 amino acids. *HcLec1* comprises an N-terminal signal peptide and a carbohydrate-recognition domain (CRD), with QPD (Gln-Pro-Asp) and MND (Met-Asn-Asp) motifs for polysaccharide binding. Tissue expression analysis showed that *HcLec1* is predominantly expressed in the gill tissue of *Hyriopsis cumingii* Lea under normal conditions, and its expression is significantly elevated in both gill and pearl sac tissues following nucleus insertion for pearl cultivation (*P* < 0.05). After immune stimulation with *Aeromonas hydrophila* and lipopolysaccharides (LPS), *HcLec1* expression levels significantly increased in both cases (*P* < 0.01), indicating a role in bivalve innate immunity. RNA interference (RNAi)-mediated knockdown of *HcLec1* led to a significant decrease in the expression levels of immune-related genes (*WAP*, *α2m*, and *Lyso*) and mineralization-related genes (*CA*, *CHS*, *Nacrein*, and *Pif*) (*P* < 0.05). In animal models for shell damage and nucleus insertion in pearl cultivation, *HcLec1* showed a consistent expression pattern, with an initial significant decrease followed by a marked increase, peaking at day 14 (*P* < 0.05). This suggests a role for *HcLec1* in pearl formation and shell repair. The recombinant HcLec1 protein demonstrated binding affinity to LPS and PGN, a robust ability to agglutinate *Escherichia coli*, *Staphylococcus aureus*, *Aeromonas veronii*, and *Aeromonas hydrophila*, and significantly inhibited bacterial growth (*P* < 0.05). Moreover, rHcLec1 promoted calcite crystal formation in saturated calcium carbonate solutions and altered crystal morphology.

**Discussion:**

The *HcLec1* gene plays a pivotal role in both innate immunity and biomineralization in the triangle sail mussel. This study enhances our understanding of the functional diversity of C-type lectins and provides a foundation for future studies on shell repair and pearl growth.

## 1 Introduction

Mollusks, inhabiting aquatic environments, are particularly susceptible to pathogen invasions (1, 2). As invertebrates, unlike fish, they rely predominantly on their innate immune system, which is distinguished by its non-specific, broad-spectrum, and swift response to pathogens (3, 4). This system targets conserved microbial elements termed pathogen-associated molecular patterns (PAMPs) rather than recognizing pathogens specifically. Upon infection, PAMPs are detected by pattern recognition receptors (PRRs), initiating the immune response (5–7). C-type lectins (CTLs), one of the most abundant families of PRRs, are particularly noteworthy in this context.

CTLs, alternatively known as C-type lectin receptors (CLRs), are calcium-dependent lectin proteins that bind carbohydrates and are ubiquitous in the animal kingdom (8). CTLs in vertebrates are notably conserved, whereas those in invertebrates exhibit considerable variation, indicative of their diverse functional roles (9–11). The CTL family is characterized by two principal polysaccharide-binding motifs: EPN (Glu-Pro-Asn)/QPD (Gln-Pro-Asp) and WND (Trp-Asn-Asp) (12). These motifs show substantial variation and are not uniformly conserved, owing to mutations. Studies indicate that *Immulectin-2* from *Manduca sexta* and *PcLec2* from *Procambarus clarkii* can activate the prophenoloxidase cascade, contributing to immune defense (13, 14). *FcLec4*, found in *Fenneropenaeus chinensis*, enhances the agglutination and elimination of *Vibrio anguillarum*, underscoring the role of CTLs as PRRs for PAMPs (15). *Cflc-1*, identified in *Chlamys farreri*, suppresses the proliferation of *E. coli* and *Candida albicans* and displays calcium-dependent agglutination of *E. coli (* 16).

*Hyriopsis cumingii* Lea is a significant pearl-producing bivalve species, ranking first globally in total pearl production (17, 18). Both the shell and pearls are products of biomineralization in pearl oysters. The repair of shell damage and the nucleus implantation process during pearl cultivation involve two key biological phenomena: (1) resistance to waterborne pathogens and the immune response triggered by nucleus implantation, and (2) guidance of calcium carbonate crystal structure formation by matrix proteins in the mantle tissue. These proteins play a critical role in the biomineralization of newly formed shells and pearls following shell damage or nucleus implantation (19, 20). This process reflects the complex interaction and regulation between immune and biomineralization functions in *H. cumingii* Lea. Following nucleus implantation, the mussel undergoes stimulation, activating immune-related genes and proteins. This response persists until pearl sac formation (wound healing), at which point the expression of mineralization-related genes and proteins changes, initiating and enhancing biomineralization functions (21, 22).

A multitude of studies underscore the potential of CTLs in the process of biomineralization. CTLs broadly refer to any proteins containing one or more C-type lectin domains (CTL domain or CRD) (8). Wilt et al. identified a CRD in the spicule matrix protein SM50 from the sea urchin *Strongylocentrotus purpuratus (* 23). They found that this CRD independently influences sea urchin mineralization and stabilizes amorphous calcium carbonate (ACC). Numerous cloned spicule matrix proteins in sea urchins contain the CRD (24). In earlier studies, a C-type lectin matrix protein, Perlucin, was isolated from the inner shell of abalone and shown to induce calcium carbonate crystal nucleation and guide crystal morphology (25). Purified Perlucin from *Haliotis discus* was also found to influence calcium carbonate crystal morphology, likely due to its high glycine and aspartic acid content, which affects the surface thermodynamics of crystal growth (26). These findings suggest that CTLs with CRDs function as matrix proteins in the biomineralization processes of sea urchins and abalones, participating in the organization and growth of calcium carbonate crystal structures. Currently, three types of CTLs have been identified in *H. cumingii* Lea. *HcLec4*, which contains four CRDs, binds to LPS and PGN, promoting early bacterial clearance through antimicrobial peptides (AMPs) (27). *HcCUB-Lec*, with a single CRD and a complement Uegf Bmp1 (CUB) domain, binds and agglutinates various bacteria, contributing to innate immunity (28). *Perlucin*, containing six conserved cysteine residues and a CRD, has been identified as a critical gene for nacre formation (29). However, at present, no studies have supported that CTLs in *H. cumingii* Lea possess both immune and biomineralization functions simultaneously.

This study identified and cloned a novel C-type lectin gene, *HcLec1*, from the mantle tissue, a critical biomineralization organ in the triangle sail mussel. HcLec1 encodes a single typical CRD and an N-terminal signal peptide. By employing gene expression profiling, constructing the HcLec1 protein expression vector, RNA interference (RNAi), Raman spectroscopy, and scanning electron microscopy, we explored the functional roles of this lectin in both innate immunity and biomineralization of shells and pearls. This research expands the molecular understanding of immunity and biomineralization processes in freshwater bivalves, providing valuable insights to improve shell repair and pearl production.

## 2 Materials and methods

### 2.1 Experimental animals and sources of materials

Triangle sail mussels were obtained from the Wuyi Aquaculture Base in Zhejiang, with an average length of 5–6 cm. They were kept in freshwater at room temperature (22–24°C) under laboratory conditions and fed *Chlorella* twice daily at 11:00 am and 11:00 pm.

Hemolymph was extracted from the hemocoel of the adductor muscle using a disposable syringe and centrifuged at 4°C and 700 × g for 10 minutes to separate the serum (supernatant) from the hemocytes (pellet). The mussel shells were opened by cutting the adductor muscle with a sterile scalpel, and tissue samples, including the mantle, gills, adductor muscle, foot, gonad, and hepatopancreas, were collected using sterile instruments. Samples were washed with sterile PBS, preserved in 1 mL of RNA preservation solution, rapidly frozen in liquid nitrogen, and stored at -80°C.

*Aeromonas hydrophila* was purchased from Beina Biological Co., while lipopolysaccharides (LPS) were obtained from Macklin Biochemical Co. *Staphylococcus aureus*, *Aeromonas veronii*, and *Escherichia coli* strains were sourced from our laboratory. Acid phosphatase assay kit and Alkaline phosphatase assay kit were purchased from Nanjing Jiancheng Bioengineering Institute.

### 2.2 Obtaining and bioinformatics analysis of full-length cDNA of *HcLec1*

The 3’ and 5’ UTRs of *HcLec1* were amplified using the SMARTER^®^ RACE 5’/3’ Kit (Takara Bio, USA) with primers listed in **Table 1**. The amplified product was purified using a gel extraction kit (Beyotime, China), then ligated into the pMD19-T vector (Takara, Japan) at 16°C for 4 h, followed by transformation into DH5α competent cells for positive clone selection. Sequencing was performed by GENEWIZ (Suzhou, China). The full-length cDNA sequence was obtained by aligning the 3’ and 5’ UTR sequences. Domain and signal peptide prediction, amino acid composition, and secondary structure prediction were carried out using the SMART platform (http://smart.embl-heidelberg.de/), the ExPASy website (https://web.expasy.org/protparam/), and the PRABI website (https://npsa-prabi.ibcp.fr/cgi-bin/npsa_automat.pl?page=/NPSA/npsa_sopma_f.html), respectively.

**Table 1:** Sequences of the primers used in this study.

Primer Name	Sequence (5’-3’)	Purpose
3’outer	CAGCACCAGCAACAACACAGAGGCA	3’RACE
3’inner	GCCTGACCTGCCCACAACTGAAGTG	
5’outer	TCCTGACCAGAAGGGGAATGTGCT	5’RACE
5’inner	CAGTTGTGGGCAGGTCAGGCATTC	
IC1-T7-F	GGATCCTAATACGACTCACTATAGGTCCAATCAATGTAAATCCTG	IC1
IC1-T7-R	GGATCCTAATACGACTCACTATAGGGACAACTCTCTCCTCCAAAA	
IC1-F	TCCAATCAATGTAAATCCTG	
ICI-R	GACAACTCTCTCCTCCAAAA	
IC2-T7-F	GGATCCTAATACGACTCACTATAGGATGCCTGACCTGCCCACAAC	IC2
IC2-T7-R	GGATCCTAATACGACTCACTATAGGCAACTCTCTCCTCCAAAACC	
IC2-F	ATGCCTGACCTGCCCACAAC	
IC2-R	CAACTCTCTCCTCCAAAACC	
IC3-T7-F	GGATCCTAATACGACTCACTATAGGAACACAGAGGCAAGGCAAAC	IC3
IC3-T7-R	GGATCCTAATACGACTCACTATAGGCATCAGGAATCATGAGGTCG	
IC3-F	AACACAGAGGCAAGGCAAAC	
IC3-R	CATCAGGAATCATGAGGTCG	
eGFP-T7-F	GGATCCTAATACGACTCACTATAGGTGGTCCCAATTCTCGTGGAAC	eGFP
eGFP-T7-R	GGATCCTAATACGACTCACTATAGGCTTGAAGTTGACCTTGATGCC	
eGFP-F	TGGTCCCAATTCTCGTGGAAC	
eGFP-R	CTTGAAGTTGACCTTGATGCC	
Clec-F	ACCTCGCTAAACCAAATG	qRT-PCR
Clec-R	ACTCTCTCCTCCAAAACC	
EF1α-F	GGAACTTCCCAGGCAGACTGTGC	
EF1α-R	TCAAAACGGGCCGCAGAGAAT	
WAP-F	TGTAATGTTGACGGGAGTG	
WAP-R	CTGTTTTGTTTTGATGGCT	
Lyso-F	CTTCTTTCTTGTTGGTCTGC	
Lyso-R	CTGGTAGTAGCCACAGGACA	
a_2_M-F	GGTGGTGATTCAAAGTCGGC	
a_2_M-R	GAAACTCGTGGTGTATTCTTGTGG	
P-Clec-F	TTGTCGACGGAGCTCGAATTCATGATCCTTGCTTATGGACCCA	Plasmid construction
P-Clec-R	GCTGATATCGGATCCGAATTCTTATGGGAATATCTGGCAAATAAATC	

### 2.3 Immune challenge, RNA extraction, and cDNA synthesis

To examine the tissue expression profile of *HcLec1* and its expression changes before and after nucleus insertion in the triangle sail mussel, six untreated mussels and six mussels 14 days post-insertion were randomly selected, and various tissues were collected. Sampling and preservation methods are described in Section 2.1. The mussels were divided into three groups: a PBS control group, an *Aeromonas hydrophila* injection group, and an LPS injection group. *A. hydrophila* was cultured overnight in broth at 37°C, centrifuged at 4000 rpm for 20 minutes, and resuspended in PBS. Approximately 1 × 10^7^ cells were injected into the adductor muscle of each mussel. For the LPS group, 100 μL of LPS diluted in PBS to a concentration of 1 mg/mL was injected into the adductor muscle, while the PBS control group was injected with 100 μL of sterile PBS. At 0, 6, 12, 24, and 48 h post-injection, six mussels from each group were randomly selected, and hemocytes, mantle, and gill tissues were collected. Sampling and preservation methods are detailed in Section 2.1. RNA was extracted using the TRIzol method. RNA concentration was measured with a NanoDrop 2000c (Thermo Fisher Scientific, USA). The extracted RNA was reverse-transcribed into cDNA using the Evo M-MLV RT Mix Kit, followed by real-time quantitative PCR analysis.

### 2.4 Real-time quantitative PCR

Quantitative analysis was conducted using TB Green^®^ Premix Ex Taq™ II (Tli RNaseH Plus) (Takara, Japan) on a Bio-Rad CFX96 system (Bio-Rad, Hercules, CA, USA) with a reaction volume of 20 μL. EF-1α was used as the reference gene, with primers listed in **Table 1**. A negative control was included for each primer pair. Relative expression level analysis was performed with 3–6 biological replicates and three technical replicates. The group with the lowest expression or the control group was used for normalization, and gene expression levels were evaluated using the 2^-ΔΔCT^ method.

### 2.5 *In vivo* RNA interference assay

Three dsRNA-HcLec1 constructs with T7 promoters at the 5’ end and one dsRNA-pEGFP construct with a T7 promoter at the 5’ end were designed. These constructs were transcribed into dsRNA using the T7 High Efficiency Transcription Kit and diluted to a concentration of 300 ng/μL. The three interference strands were designated as Chain 1, Chain 2, and Chain 3. The triangle sail mussels were divided into a negative control (NC) group and a dsRNA-HcLec1 injection group. Injections of 100 μL EGFP and 100 μL dsRNA-HcLec1 were administered into the adductor muscle of the mussels. After 24, 48, and 72 h post-injection, mantle tissue samples were collected. qRT-PCR and the 2^-ΔΔCT^ method were used to assess knockdown efficiency, with normalization against the negative control group.

### 2.6 Construction of recombinant plasmids and protein induction *in vitro*

The CDS region of *HcLec1* was ligated into the pET-32a plasmid (without the thrombin site) (from our laboratory) using a seamless cloning kit via the EcoRI restriction site. The recombinant plasmid was transformed into competent Origami (DE3) cells, which were cultured at 37°C, 200 rpm until the OD600 reached 0.6–0.8. IPTG was then added to a final concentration of 0.5 mM, and the cells were induced overnight at 16°C, 180 rpm. SDS-PAGE gel electrophoresis was performed to assess the induction of recombinant protein expression. The induced protein was subsequently purified using a His-Tag Protein Purification Kit (Beyotime, China) and analyzed by SDS-PAGE. The recombinant *HcLec1* proteins are referred to as rHcLec1 throughout the study.

### 2.7 Western blot

After SDS-PAGE, the protein bands were transferred onto a methanol-activated PVDF membrane using a semi-dry transfer method. The PVDF membrane was then blocked with 10% non-fat milk diluted in TBST at room temperature for 1 h, followed by incubation with a 1:1000 dilution of mouse anti-His tag monoclonal antibody (Beyotime, China) at 4°C overnight. After three washes with TBST, the membrane was incubated with a 1:1000 dilution of HRP-conjugated goat anti-mouse IgG secondary antibody at room temperature for 1 h. Following three additional washes with TBST, the membrane was developed, and detection was performed using a versatile gel imaging system (Bio-Rad, Hercules, CA, USA).

### 2.8 Detection of binding activity of rHcLec1 and PAMPs

The binding activity of rHcLec1 with LPS and peptidoglycan (PGN) was assessed using an ELISA kit. In a 96-well plate, 5 μL of LPS and PGN at a concentration of 80 μg/mL were added to each well and incubated at 37°C overnight. Subsequently, 50 μL of 2% BSA (1 mg/mL) was added to each well and incubated at room temperature for 2 h, followed by five washes with TBS. In the negative control group, 50 μL of rTrx (recombinant Thioredoxin) was added, while 50 μL of rHcLec1 protein was added to the experimental group, and incubation occurred at 37°C for 2 h. Each well was then treated with 100 μL of anti-His-tag mouse monoclonal antibody (1:1000 dilution) and 100 μL of HRP-conjugated sheep anti-mouse secondary antibody (1:1000 dilution), incubated at 37°C for 2 h, and washed five times with TBS. Color development was achieved by adding 100 μL of TMB substrate solution for 30 minutes, followed by 100 μL of TMB stop solution. Absorbance was measured at 450 nm. A result was considered positive if (P [sample] - B [blank])/(N [negative] - B [blank]) > 2.1. Each experiment was performed in triplicate.

### 2.9 Bacterial inhibition test

*Staphylococcus aureus*, *Escherichia coli*, *Aeromonas veronii*, and *Aeromonas hydrophila* were cultured to the logarithmic growth phase (15) and diluted to a concentration of 1 × 10^4^ CFU/mL in TBS solution, followed by inoculation into a 96-well plate. For the experimental group, 50 μL of rHcLec1 (200 μg/mL) was added to 50 μL of bacterial solution, while the control group received 50 μL of TBS solution. The mixtures were incubated at 37°C for 2 h. Subsequently, 20 μL of each mixture was transferred to a nutrient broth medium for further incubation. OD600 absorbance values were measured at 12 h using a microplate reader.

### 2.10 Bacterial agglutination test

Bacteria in the logarithmic growth phase were collected for Giemsa staining, and the stained microorganisms were suspended in TBS buffer. A 10 μL aliquot of the microbial suspension was incubated with 25 μL of rHcLec1 (200 μg/mL) dissolved in TBS buffer or with 25 μL of TBS buffer (control group) at room temperature for 1 h. Observations were made using an optical microscope.

### 2.11 *In Vitro* growth of crystals in the presence of rHcLec1

A saturated calcium carbonate solution was prepared following the method of Zhenguang Yan et al. (30). With continuous stirring, 30 mL of 100 mM sodium bicarbonate was added dropwise to 120 mL of 40 mM calcium chloride solution until the solution became cloudy, at which point stirring was immediately stopped. The pH was adjusted to 8.2 with NaOH, and the solution was filtered through a 0.22 μm filter. To assess the effect of rHcLec1 on calcite crystal morphology *in vitro*, the recombinant protein was incubated with the prepared saturated calcium carbonate solution at 4°C for 48 h. Crystals were examined using scanning electron microscopy, and their crystal forms were analyzed by Raman spectroscopy. The experiment included a blank control group (300 μL saturated calcium carbonate solution only), a negative control group (300 μL saturated calcium carbonate solution with protein elution buffer), and an experimental group (300 μL saturated calcium carbonate solution with 50 μL of rHcLec1 at 1.0 mg/mL).

### 2.12 Statistical analysis

The graphs were plotted using GraphPad Prism 9. Data analysis was performed using ANOVA and the t-test in SPSS 26.0. Tukey’s test was used for multiple comparisons, with P < 0.05 indicating significant differences.

## 3 Results

### 3.1 Cloning and sequence analysis of *HcLec1*

**Figure 1:**
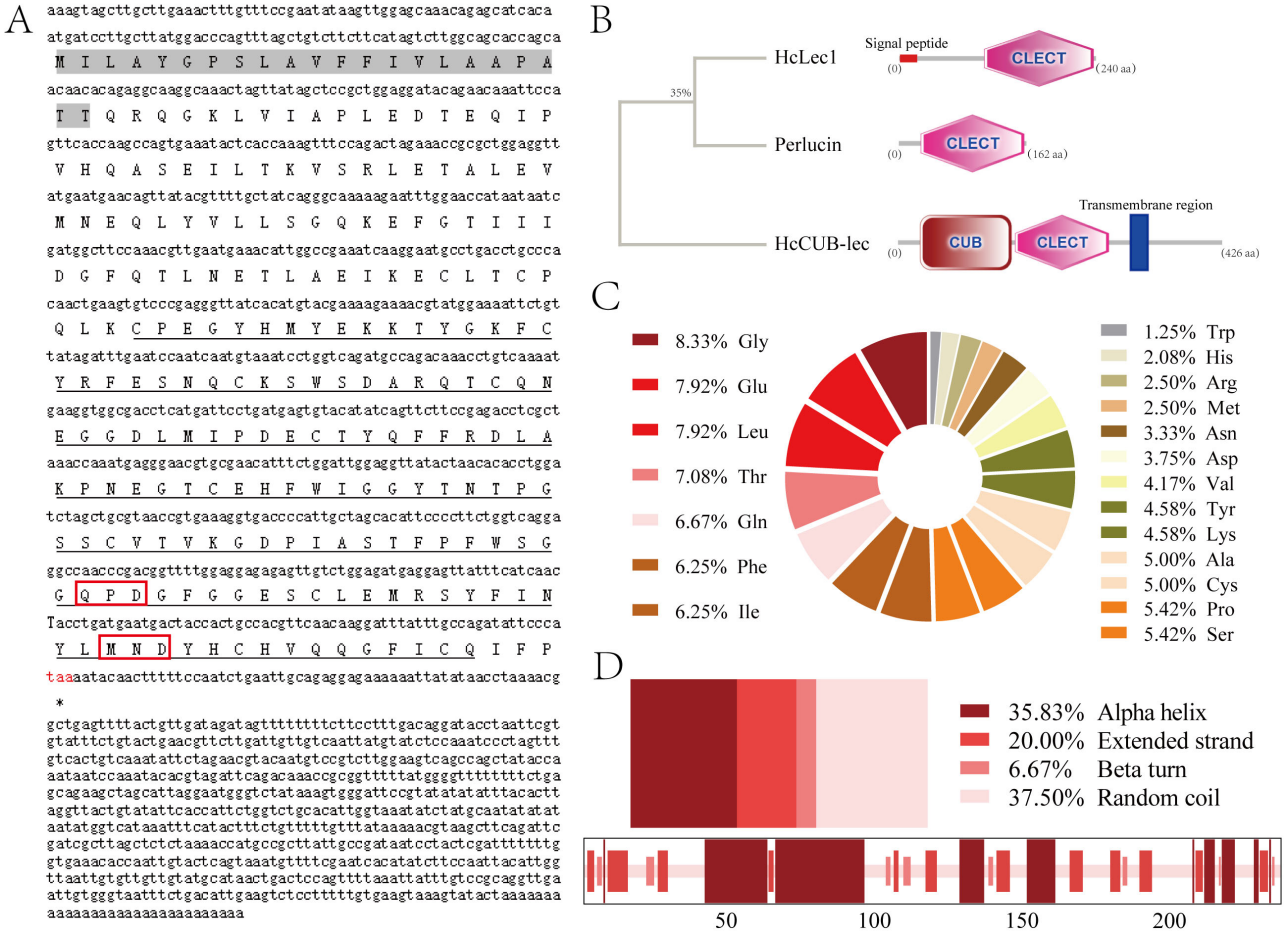
Bioinformatic analysis of *HcLec1*. **(A)** Full-length *HcLec1* gene obtained by cloning. The gray background indicates the signal peptide, the black dashed line represents the conserved domains, the red box highlights the polysaccharide-binding motifs, and the red “taa” indicates the stop codon. **(B)** Structural domain prediction of HcLec1 and comparison of HcLec1 with other CTL sequences from *H. cumingii* Lea. The “CLECT” denotes the C-type lectin domain. **(C)** Amino acid composition of the HcLec1 protein. **(D)** Proportions of secondary structure elements in the HcLec1 protein.

Cloning results revealed that the full-length cDNA of *HcLec1* is 1552 bp (**Figure 1A**), with a 3’ UTR of 770 bp, a 5’ UTR of 59 bp, and a CDS of 723 bp, encoding 240 amino acids. Among these, Glycine (Gly) accounts for 8.3%, followed by Glutamic acid (Glu) and Leucine (Leu) at 7.9% each (**Figure 1C**). The structure of HcLec1 is different from that of previous CTLs in *H. cumingii* Lea. SMART analysis predicted that the HcLec1 protein contains an N-terminal signal peptide and a carbohydrate recognition domain (CRD) with QPD (Gln-Pro-Asp) and MND (Met-Asn-Asp) motifs for polysaccharide binding (**Figure 1B**). The predicted molecular weight of the HcLec1 protein is 27.03 kDa, with a secondary structure comprising 37.5% random coils and 35.83% alpha helices (**Figure 1D**). The GenBank accession number is PP056159.

### 3.2 Expression profile of *HcLec1* in various tissues of *H. cumingii* Lea

**Figure 2:**
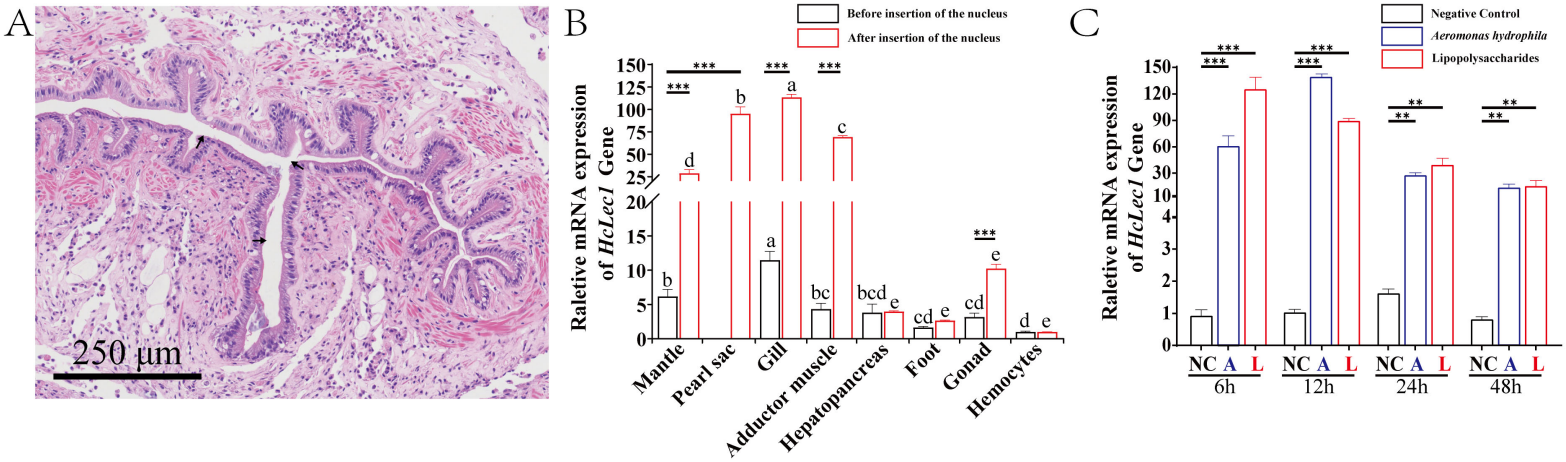
*HcLec1* tissue expression profile and challenge assay. **(A)** HE-stained schematic showing pearl sac formation after nucleus implantation, with black arrows indicating the cavity formed by the pearl sac. **(B)** Relative expression levels of *HcLec1* in various tissues of untreated and nucleus-implanted mussels. Different lowercase letters indicate significant differences between tissues within the same group (*P* < 0.05). **(C)** Relative expression levels of HcLec1 following bacterial challenge. NC represents the negative control group (injected with sterile PBS), while A and L represent the A. hydrophila and LPS injection groups, respectively. ** denotes highly significant differences (*P* < 0.01), and *** denotes extremely significant differences (*P* < 0.001).

The qPCR results demonstrated that *HcLec1* is expressed in all tissues of *H. cumingii* Lea, with significantly higher relative expression levels observed in the gill tissues of untreated mussels compared to other tissues, followed by the mantle (*P* < 0.05, **Figure 2B**). Fourteen days after the nucleus implantation is a critical period for the formation of the pearl sac. During this time, the smooth muscle in the mantle undergoes specialization into columnar epithelial cells, forming a cavity (the pearl sac) that encloses the foreign object. The specialized pearl sac tissue at this stage is crucial for the study of biomineralization (**Figure 2A**). The quantitative analysis revealed that *HcLec1* expression levels were significantly elevated (*P* < 0.01) in the gill tissue, mantle tissue, pearl sac (specialized mantle tissue), adductor muscle, and gonads (**Figure 2B**). Specifically, following nucleus implantation, the relative expression levels of *HcLec1* in the mantle, gills, adductor muscle, and gonads increased approximately 4.6-, 10-, 17-, and 3-fold, respectively, with no significant changes detected in the hepatopancreas, foot, or hemocytes (*P* > 0.05). Although hemocyte-mediated immunity is a crucial component of innate immunity in mollusks, our results revealed that *HcLec1* exhibited the lowest relative expression in hemocytes. These findings suggest that *HcLec1* may primarily play an immune role in the gills, while exerting a biomineralization function in the mantle and pearl sac.

### 3.3 *A. hydrophila* and LPS stimulated *HcLec1* expression

The relative expression of *HcLec1* increased sharply and significantly following stimulation with *A. hydrophila* and LPS (*P* < 0.01), peaking at 6 h and 12 h post-stimulation. Expression levels significantly decreased at 24 h and 48 h but remained substantially higher than those of the control group (*P* < 0.01, **Figure 2C**). Under *A. hydrophila* and LPS stimulation, *HcLec1* expression increased approximately 60-fold and 120-fold compared to the control group at 6 h and about 140-fold and 90-fold at 12 h. By 24 h, expression sharply decreased (*P* < 0.01), and at 48 h, expression levels further declined but remained 14–15 times higher than the NC group (*P* < 0.01). These findings suggest that *HcLec1* is rapidly upregulated upon stimulation with *A. hydrophila* and LPS, with peak expression occurring earlier following LPS stimulation compared to *A. hydrophila*.

### 3.4 *HcLec1* regulates immune and mineralization-related genes and enzyme activities

**Figure 3:**
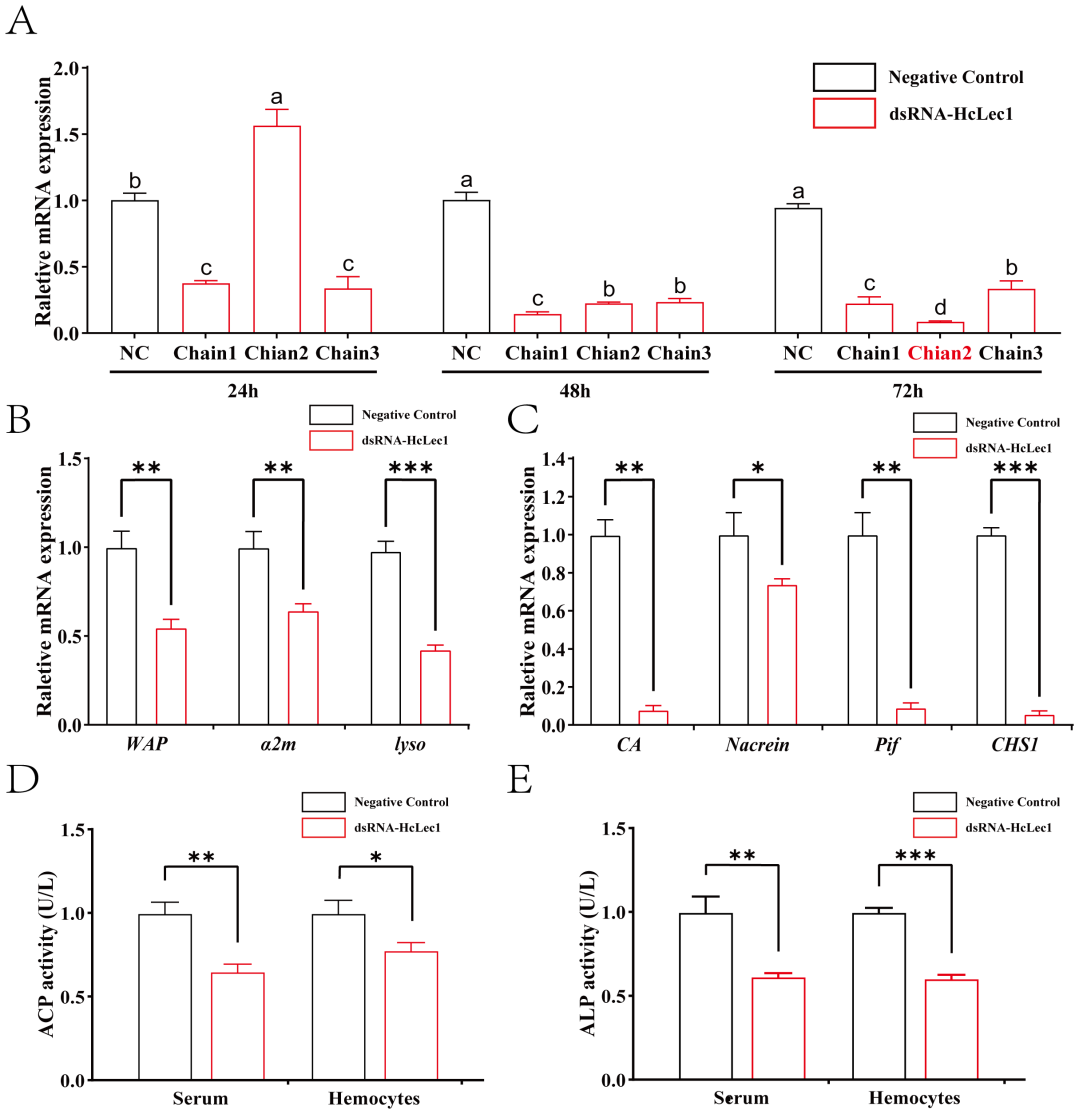
*HcLec1* RNAi Experiment. **(A)** Effects of three dsRNA-HcLec1 sequences. NC represents the negative control group (injected with eGFP), while Chain1, Chain2, and Chain3 correspond to the experimental groups (each injected with one of the three dsRNA-HcLec1 sequences). Different lowercase letters indicate significant differences between the same groups at different times (*P* < 0.05). **(B, C)** display relative expression changes in immune- and mineralization-related genes following *HcLec1* knockdown. **(D, E)** depict alterations in ACP and ALP enzyme activities after HcLec1 knockdown. Black bars represent the control group (injected with eGFP), and red bars represent the experimental group (injected with dsRNA-HcLec1). * indicates *P* < 0.05, ** indicates *P* < 0.01, and *** indicates *P* < 0.001.

The results of *HcLec1* relative expression after RNAi demonstrated that all three dsRNAs achieved significant knockdown efficiency at 48 h and 72 h (*P* < 0.001), with Chain 2 showing a 90% knockdown at 72 h (**Figure 3A**). Consequently, Chain 2 was selected for subsequent RNAi experiments. Interference with *HcLec1* using Chain 2 significantly reduced the expression levels of immune-related genes *WAP*, *α2m*, and *Lyso* (*P* < 0.01, **Figure 3B**). *CA*, *CHS*, *Nacrein*, and *Pif* are key matrix proteins in *H. cumingii* Lea (30–32). After *HcLec1* knockdown, the relative expression levels of mineralization-related genes *CA*, *CHS*, and *Pif* were significantly reduced by 10–20 fold (*P* < 0.01, **Figure 3C**), while *Nacrein* expression decreased by 30% (*P* < 0.05). ACP and ALP activities are crucial for biomineralization (33). This study revealed that ACP and ALP enzyme activities were significantly downregulated following *HcLec1* interference (*P* < 0.05). ACP activity decreased by 35% in serum and 22% in hemocytes (**Figure 3D**), whereas ALP activity showed a significant 40% reduction in both serum and hemocytes (**Figure 3E**). These findings suggest that *HcLec1* knockdown adversely impacts both immune and biomineralization processes in *H. cumingii* Lea.

### 3.5 *HcLec1* involvement in shell and pearl mineralization processes in *H. cumingii* Lea

**Figure 4:**
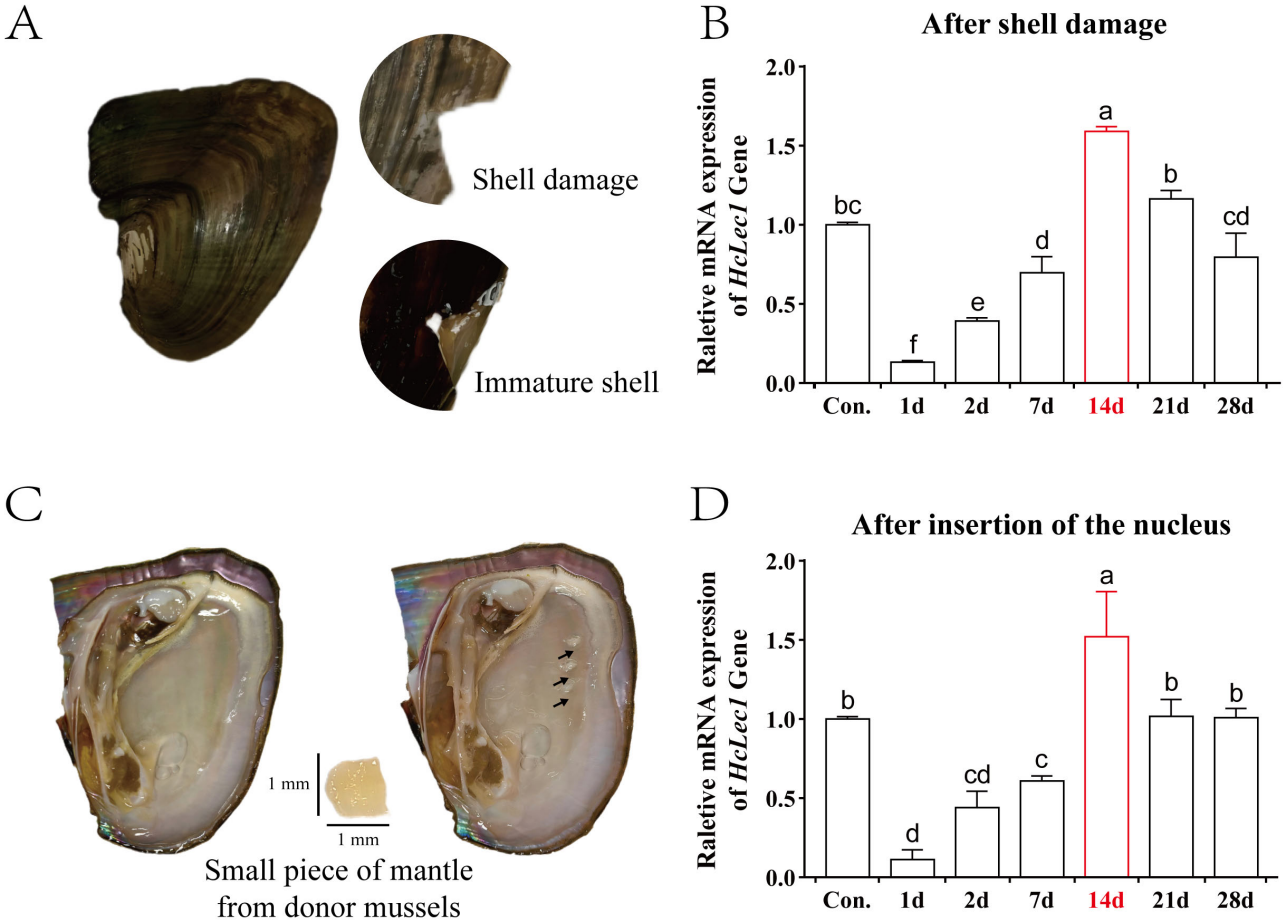
Relative Expression Changes of *HcLec1* in *H. cumingii Lea* During Mineralization. **(A)** Procedure and observations of the shell damage experiment in *H. cumingii Lea*. **(B)** Relative expression trend of *HcLec1* during shell repair following damage. **(C)** Nucleus insertion procedure in *H. cumingii Lea*, with the black arrow indicating the insertion site. **(D)** Relative expression trend of *HcLec1* after nucleus insertion, where "Con." represents the control group at day 0. Different lowercase letters indicate significant differences between groups (*P* < 0.05).

In the shell damage experiment, cracks in the shell of *H. cumingii* Lea gradually develop into a thin membrane (immature shell (34)) over time, which subsequently mineralizes and heals (**Figure 4A**). Upon inserting a small piece of mantle tissue from the donor mussel (known as “saibo”), the recipient mantle is stimulated to form a pearl sac, which envelops the tissue and secretes nacre, leading to pearl formation (**Figure 4C**). Results show that during both shell repair and “saibo” insertion, *HcLec1* expression follows a similar pattern: it decreases significantly initially and then rises steadily to reach control levels (*P* < 0.05, **Figure 4B, D**). Compared to the control, *HcLec1* expression was significantly reduced on day 1 and remained lower than control levels from days 1 to 7 (*P* < 0.05). By day 14, *HcLec1* expression significantly increased to 1.5–1.6 times the control level. Days 10 to 15 represent the phase of membrane formation and subsequent hardening at the shell damage site, as well as the initial formation of the pearl sac at the insertion site. On days 21 and 24, *HcLec1* expression levels declined and stabilized, showing no significant difference from the control group (*P* > 0.05). These findings suggest that *HcLec1* plays a role in both shell repair and pearl sac formation processes in *H. cumingii* Lea.

### 3.6 Recombinant HcLec1 protein inhibits bacterial growth and agglutinates bacteria by recognizing PAMPs

**Figure 5:**
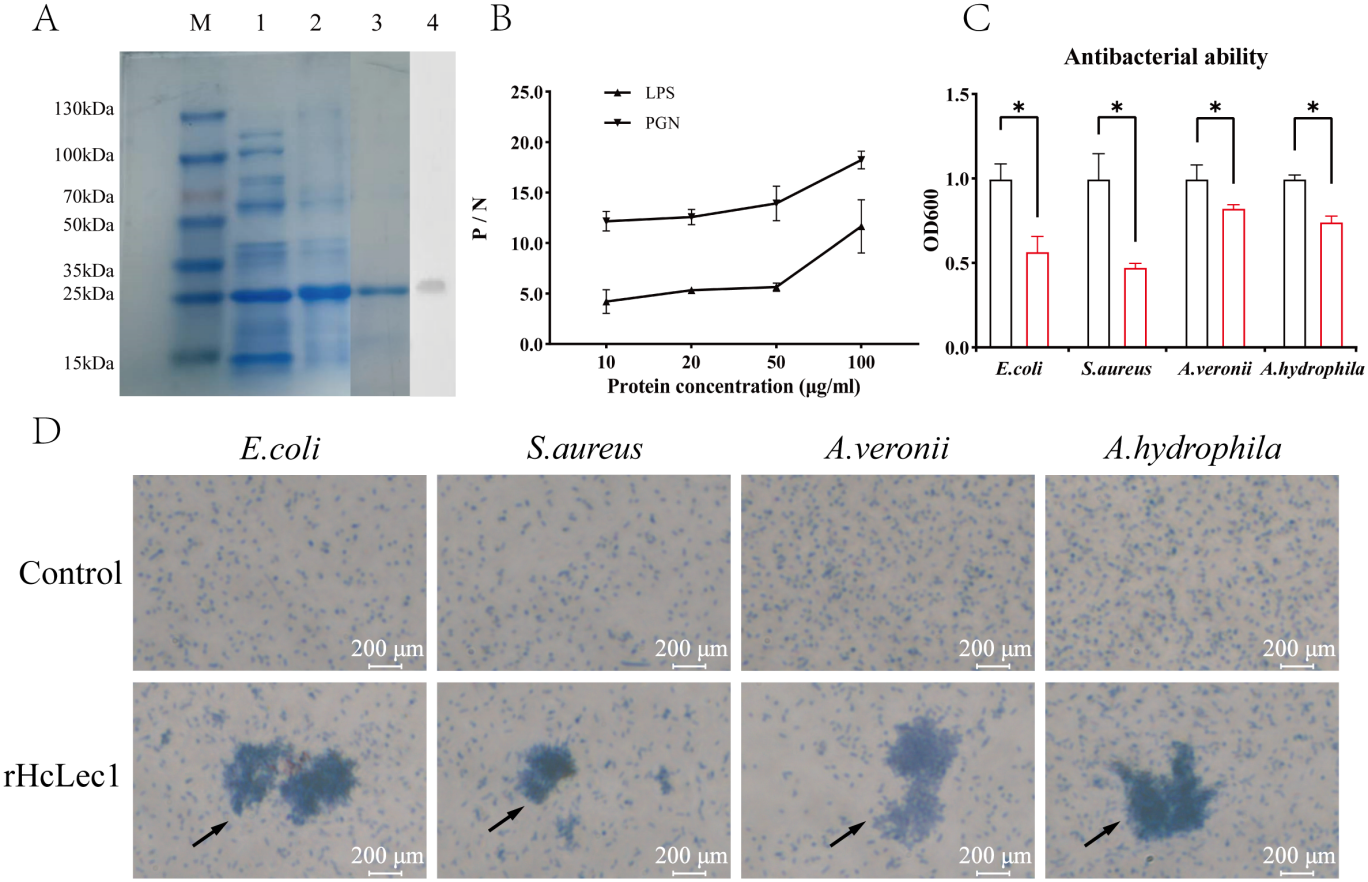
Prokaryotic Expression and Antibacterial Activity Analysis of rHcLec1. **(A)** SDS-PAGE and Western blot validation of rHcLec1. Lane M: Marker; Lane 1: Expression of rHcLec1 in the supernatant; Lane 2: Expression of rHcLec1 in the precipitate; Lane 3: Fourth elution of rHcLec1; Lane 4: Western blot validation results. **(B)** Binding activity of rHcLec1 to PGN and LPS. **(C)** Antibacterial effect of rHcLec1, with red bars representing the experimental group (200 μg/mL of rHcLec1) and black bars representing the control group (equivalent concentration of TBS). **(D)** Agglutination effect of rHcLec1 on *S. aureus*, *E. coli*, *A. veronii*, and *A. hydrophila*. The experimental group (rHcLec1, 200 μg/mL) is labeled as "rHcLec1," and the control group (TBS at the same concentration) as "Control." Black arrows indicate areas of significant agglutination. * indicates *P* < 0.05.

To investigate the response of HcLec1 protein to bacteria *in vitro*, we successfully induced the recombinant HcLec1 protein (referred to as rHcLec1, **Figure 5A**). The results indicated that rHcLec1 can recognize and bind both LPS and PGN, with a higher binding affinity for PGN compared to LPS (**Figure 5B**). This finding supports the role of rHcLec1 as a PRR that binds to PAMPs. PGN, a key component of the cell walls in both Gram-positive and Gram-negative bacteria, suggests that rHcLec1 has broad-spectrum antibacterial activity, allowing it to recognize both bacterial types. Bacterial inhibition assays showed that rHcLec1 significantly suppressed the growth of *E. coli*, *S. aureus*, *A. veronii*, and *A. hydrophila*, reducing their growth rates by 43%, 53%, 18%, and 26%, respectively, when added to the growth medium (**Figure 5C**). Giemsa staining revealed a clear agglutination effect of rHcLec1 on these four bacterial species (**Figure 5D**). This suggests that, as a C-type lectin, rHcLec1 may inhibit bacterial growth not only by activating immune-related genes but also by directly inducing bacterial agglutination.

### 3.7 rHcLec1 promotes calcite formation *in vitro*

**Figure 6:**
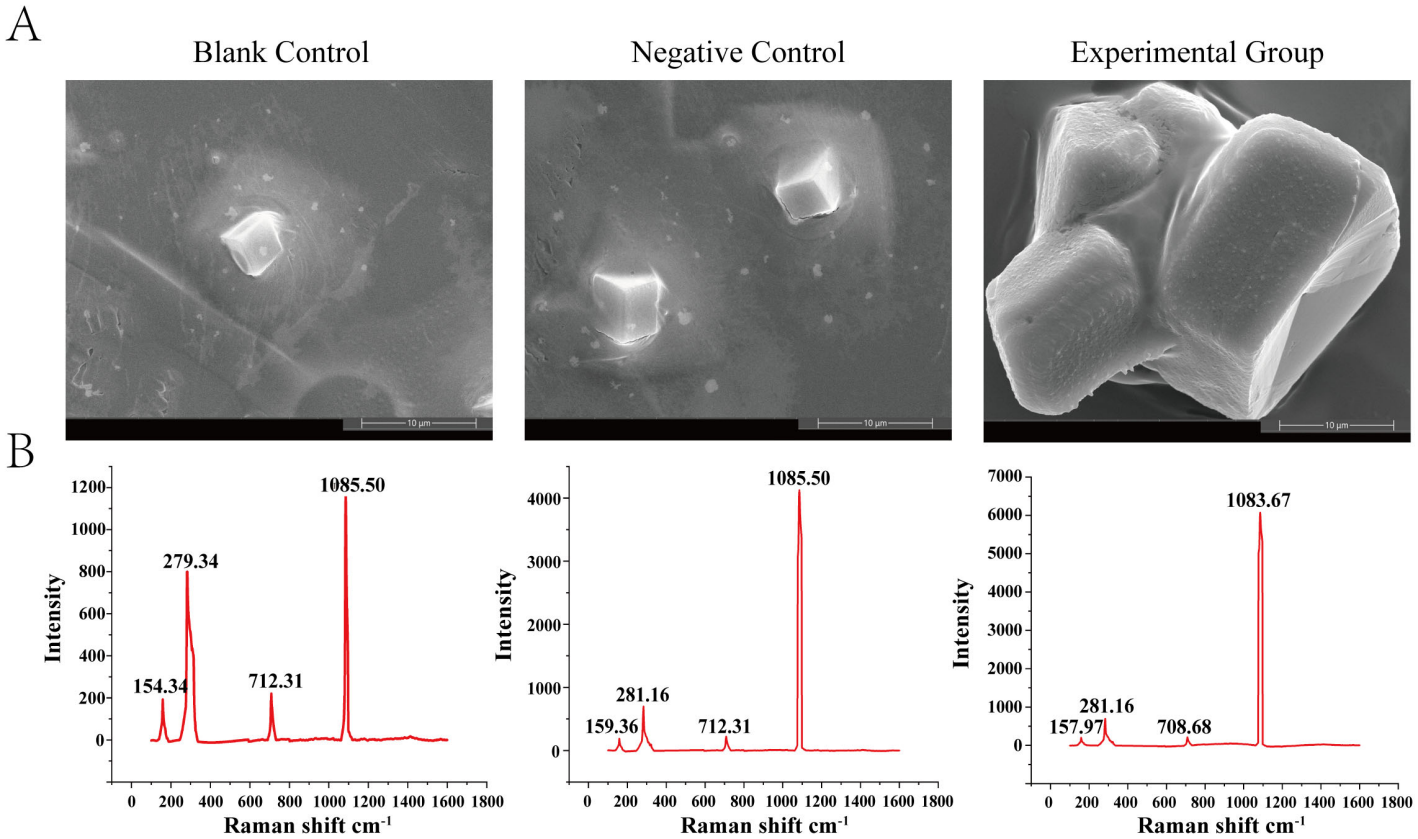
Role of rHcLec1 in the in vitro crystallization of calcium carbonate. **(A)** Results from scanning electron microscopy. **(B)** Corresponding Raman spectroscopy results. The Blank Control contains only a saturated calcium carbonate solution, the Negative Control includes protein elution buffer, and the Experimental Group includes 50 μL of rHcLec1 (1.0 mg/mL).

The mineralization capacity of bivalves is primarily reflected in calcium carbonate deposition and the regulation of the morphology and properties of calcium carbonate crystals. After co-culturing at 4°C for 48 h, rectangular crystals were observed in the solutions of both the blank and negative control groups (**Figure 6A**). Compared to the control group, crystal growth was significantly enhanced in the presence of rHcLec1. At the same magnification, crystals formed under the influence of rHcLec1 protein were several times larger than those in the control group and exhibited significant fusion. Raman spectroscopy analysis of the crystals indicated nearly identical spectral bands across all three groups (**Figure 6B**). Two main bands were identified at 279.34–281.16 and 1083.67–1085.50, along with two weaker bands at 154.34–159.36 and 708.68–712.31, which are characteristic of calcite (35–37). These results suggest that rHcLec1 can promote calcite formation and fusion.

## 4 Discussion

C-type lectins are among the largest families of pattern recognition receptors (PRRs), activating the immune system against invading pathogens through the recognition of pathogen-associated molecular patterns (PAMPs). They are widely distributed in both vertebrates and invertebrates; however, in invertebrates, they are generally not conserved. This structural diversity has resulted in a wide array of functions within the CTL family in invertebrates. In mollusks, CTLs have been reported to serve various functions. Beyond their well-known roles in innate immunity (38), recognizing and binding to PAMPs (39), and agglutinating bacteria (40), they had also been shown to perform many other functions. In the echinoderm *Strongylocentrotus purpuratus*, it was found that sea urchin spicule matrix proteins, such as SpSM29, SpC-lectin, SM30, and PM27, contain CTL carbohydrate recognition domains (CRDs), which are involved in post-spicule formation (23, 24). In mollusks, a CTL named *MeML* was found in the labial palps of *Crassostrea virginica* and *Mytilus edulis*, and it may be involved in the selection of food particles in suspension-feeding bivalves (41, 42). In *Ruditapes philippinarum*, CTLs are thought to have potential functions in responding to cold stress (43). Additionally, Perlucin, a purified CTL from *Haliotis discus* and *H. cumingii* Lea, has been shown to play a role in altering the morphology of calcium carbonate crystals and in nacre formation (26, 29).

In this study, a C-type lectin (HcLec1) from the freshwater pearl mussel *H. cumingii* Lea was isolated, identified, and cloned. Structurally simple, *HcLec1* contained only a signal peptide and a CRD, making it suitable for functional studies as a representative C-type lectin. The results of this study demonstrated that *HcLec1* was expressed across various tissues, with the lowest expression in hemocytes. This finding was similar to those for *Perlucin* in *Haliotis discus discus* and *FcLec4* in *Fenneropenaeus chinensis (* 15, 26), suggesting tissue-specific expression of CTLs (44, 45). Huang et al. found that the mantle of *Pinctada fucata* contains a high concentration of CTLs, with more immune genes expressed in the mantle than in hemocytes, a pattern similar to that of *HcLec1 (* 46). The gill tissue in *H. cumingii* Lea is a key site for exchange with the external water environment and for pathogen defense (16, 47, 48). Thus the high expression of *HcLec1* in the gill implied its potential role in innate immunity.

The CRD of CTLs contained carbohydrate-binding motifs that were critical for recognizing and binding to PAMPs (29, 39, 49). The CRD of *HcLec1* contained two carbohydrate-binding motifs (QPD and MND), and results from injection stimulation and ELISA indicated that these motifs responded to *A. hydrophila* and LPS and could bind to both LPS and PGN. LPS and PGN are representative PAMPs of Gram-negative and Gram-positive bacteria, respectively (7). Our findings also showed that rHcLec1 could directly agglutinate Gram-positive bacteria (*S. aureus*) and Gram-negative bacteria (*E. coli*, *A. veronii*, and *A. hydrophila*) *in vitro*, without the addition of Ca^2+^, indicating that rHcLec1’s agglutination function is calcium-independent. This result was consistent with the functional studies of *SPL-1* in *Saxidomus purpuratus* and *CfLec-2* in *Chlamys farreri* (50, 51). Additionally, *HcLec1* was found to directly inhibit the growth of these four bacterial species *in vitro*, suggesting that *HcLec1* not only enhanced bacterial phagocytosis through complement system activation *in vivo* but also directly bound to and agglutinates bacteria, affecting their proliferation.

The pearl formation process in *H. cumingii* Lea can be divided into three main stages: an initial immune rejection stage following the implantation of a small mantle tissue piece from the donor mussel into the recipient’s mantle, a stage where the connective tissues of both fuse and specialize into a pearl sac, and finally, a mineralization stage where nacre is secreted and deposited to form the pearl (52, 53). Throughout this process, both the mantle and pearl sac serve as critical mineralizing tissues in *H. cumingii* Lea, with the pearl sac being particularly essential for pearl cultivation (21, 54, 55). The significant expression of *HcLec1* in both the mantle and pearl sac suggested that *HcLec1* may play roles not only in immunity but also in mineralization. Our results indicated that the expression pattern of *HcLec1* aligned with the mineralization process during nucleus implantation and shell self-repair. The 10–14 day period is critical for pearl sac and shell repair, as the pearl sac nearly forms, and new shell material is deposited, marking a peak in mineralization activity (29, 56, 57).

CTLs have been shown to participate in *in vitro* mineralization and guide crystal formation. The protein Perlucin from *Haliotis discus discus* and *Haliotis laevigata* has been demonstrated to regulate crystal formation by either promoting or inhibiting calcium carbonate crystal formation and controlling crystal morphology and orientation (25, 26, 58, 59). Our study corroborated these findings: rHcLec1 was observed to promote calcite formation *in vitro* and facilitate the fusion of multiple calcite crystals into a larger, more complex structure. Morphologically, the resulting crystals appeared as a larger aggregate formed by the fusion of several smaller crystals, with a connecting and fused region between them. Although we did not investigate further whether this fusion is due to rHcLec1 producing a glue-like substance or embedding multiple crystals together, previous research has shown that P60 in *Pinctada fucata* can bind crystals together, suggesting that rHcLec1 may have a similar function in crystal aggregation (60). The formation of pearls depends on the combined action of foreign materials, calcium carbonate, and proteins (61). Many proteins can randomly or directionally regulate the formation of crystal morphology and size. Currently, the cultivation and harvesting of pearls cannot be controlled artificially. Therefore, protein-mediated formation of high-quality pearls may be a future research direction (62, 63). rHcLec1, as a protein identified from *H. cumingii* Lea with dual functions in immunity and biomineralization, could provide new molecular materials and a foundation for forming high-quality pearls by influencing crystal formation.

In summary, we demonstrated that *HcLec1* in *Hyriopsis cumingii* Lea participated in innate immunity, could agglutinate and inhibit bacterial growth *in vitro*, promoted calcite crystal formation and fusion, and played a significant role in the biomineralization process of the mussel. This study may provide a molecular basis for shell repair and pearl growth, offering new materials and insights for biomineralization applications.

## Data Availability

The datasets presented in this study can be found in online repositories. The names of the repository/repositories and accession number(s) can be found below: https://www.ncbi.nlm.nih.gov/nuccore/2647031310, nuccore/2647031310.
